# Validation of diagnostic ICHD-3 criteria for menstrual migraine

**DOI:** 10.1177/03331024221099031

**Published:** 2022-05-06

**Authors:** Iris E Verhagen, Hermes AJ Spaink, Britt WH van der Arend, Daphne S van Casteren, Antoinette MaassenVanDenBrink, Gisela M Terwindt

**Affiliations:** 1 Department of Neurology, Leiden University Medical Center, Leiden, The Netherlands; 2 Department of Internal Medicine, Erasmus University Medical Center, Rotterdam, The Netherlands

**Keywords:** Headache classification, ICHD-3, menstrual migraine, women, E-diary

## Abstract

**Objective:**

To assess validity of ICHD-3 diagnostic criteria for menstrual migraine.

**Methods:**

We performed a longitudinal E-diary study in premenopausal women with migraine. Menstrual migraine diagnosis was self-reported at baseline, and verified according to diary based ICHD-3 criteria and a previous proposed statistical model. Validity of self-reported menstrual migraine was compared to diary based diagnosis and statistical diagnosis. Test-retest reliability and concordance between both methods were determined. Clinical characteristics of perimenstrual and non-perimenstrual migraine attacks were compared in women with and without menstrual migraine.

**Results:**

We included 607 women. Both women who did and women who did not self-report to suffer from menstrual migraine fulfilled ICHD-3 criteria in the E-diary in two thirds of cases. Pure menstrual migraine was extremely rare (<1%). Concordance between statistical and diary based diagnosis was minimal (κ = 0.28, 95% CI:0.23–0.33). Women diagnosed with menstrual migraine showed 37–50% longer attack duration and increased triptan intake (OR 1.19–1.22, p < 0.001) during perimenstrual attacks.

**Conclusion:**

Self-reported menstrual migraine diagnosis has extremely poor accuracy. Two thirds of women suffer from menstrual migraine, independent of self-reports. Pure menstrual migraine is rare. Women with menstrual migraine have longer attack duration and increased triptan intake during perimenstrual attacks, in contrast to women without menstrual migraine. Prospective headache (E-)diaries are required for a menstrual migraine diagnosis, also in clinical practice.

## Introduction

Menstruation is the most reported trigger factor for migraine in women (1). The occurrence of migraine attacks is most likely during the five-day window surrounding the start of the menstruation (days −2 to +3 of the menstrual cycle) (2–5). Perimenstrual attacks are suggested to be more severe with longer duration, and with more recurrences after triptan intake (3,6–11), but an association between migraine and menstruation seems not to be present in all women (12,13).

The International Classification of Headache Disorders (ICHD-3) provides criteria for menstrual migraine (MM) in the appendix, which is defined as the occurrence of migraine attacks during the menstrual window in at least two out of three menstrual cycles (14). A distinction is made between pure MM, when attacks occur exclusively during the menstrual window, and menstrually-related migraine, when attacks also occur outside of this window.

Criteria for MM have not been sufficiently validated and are, therefore, part of the ICHD-3 appendix. Indeed, several aspects of the criteria still require a more extensive scientific basis before they can be incorporated into the main classification. According to the current criteria, a diagnosis based on three months of prospective headache diaries is recommended for research purposes but deemed not strictly necessary for clinical practice. However, earlier research has emphasised the overall necessity of headache diaries, as patients do not reliably recall headache-related information (15,16).

Furthermore, it is still disputed whether the ‘two out of three menstrual cycles’ criterion has sufficient sensitivity and specificity (16,17). Women with a high attack frequency are by definition more likely to receive an MM diagnosis, while women with a very low attack frequency will not be diagnosed with MM, despite a clear association. A mathematical model for diagnosing statistical menstrual migraine (sMM) was recently proposed with the purpose of excluding these spurious associations between migraine and menstruation in women with high attack frequencies and to enable diagnosis in women with only few attacks (17,18). This statistical approach was shown to have a higher specificity than the original ICHD-3 criteria in a population of 165 women with migraine, but performance in other, larger patient samples still needs to be determined.

The aim of this current study was to assess validity of the ICHD-3 appendix criteria for MM in a large female migraine population. In addition, concordance between IHCD-3 criteria and sMM was determined. Lastly, we distinguished women with and without MM, and clinical characteristics of perimenstrual and non-perimenstrual attacks were compared between these groups.

## Methods

This longitudinal cohort study was conducted as part of the Leiden University Migraine Neuro Analysis (LUMINA) program at the Leiden Headache Center (19). Data were collected between August 2018 and July 2021. Ethical consideration for this study was obtained from the Medical Ethics committee of the LUMC, who judged that the study was not associated with ethical concerns. Therefore, patients did not have to provide informed consent for this study specifically. All data were analysed fully anonymised.

Dutch female migraine patients aged ≥18 years were recruited and were considered eligible after a two-step inclusion process using validated questionnaires. Patients were asked to fill out a web-based screening questionnaire with a sensitivity of 0.93 and specificity of 0.36 for diagnosing migraine (20). Patients who fulfilled the screening criteria, were sent an extended questionnaire with a specificity of 0.95 and sensitivity of 0.45 (14,19). Final diagnoses were made after a clinical interview by a neurology resident with consultation of a headache specialist (GT) or a researcher with headache expertise (IV, BvdA, DvC).

For the current study we considered women diagnosed with migraine with a natural menstrual cycle or using combined oral contraceptive pills, provided that they included hormone free intervals (regardless of the frequency). Postmenopausal, pregnant or breast-feeding women were excluded.

Women with chronic migraine (CM) were not excluded, as current ICHD-3 criteria do not explicitly state that women with CM should not be diagnosed with MM. CM was nonetheless assessed in each patient, and was defined according to the ICHD-3 criteria as three consecutive months with ≥15 headache days and ≥8 migraine days per month (14). In addition, medication overuse headache (MOH) was defined as three consecutive months with ≥10 acute medication days per month (≥10 for triptans, ≥15 for analgesics, ≥10 for combination of acute medications) (14). A month was defined as a period of 28 days.

At baseline, patients were asked whether their migraine attacks were associated with the menstrual cycle. Subsequently, they were followed prospectively with previously validated E-diaries during at least three menstrual cycles (17). Patients provided daily information about presence of headache, its characteristics and accompanying symptoms, aura symptoms, use of acute (pain) medication and menstrual bleeding. The E-diary was time-locked, i.e. no longer accessible after 48 hours. Patients were encouraged to respond on each day through daily e-mails and text message reminders. Compliance to the E-diary had to be at least 80%. Days with missing data were considered headache free. An underlying algorithm verified for each headache day whether the ICHD-3 criteria for migraine were met (14). In addition, days with aura symptoms lasting for 5–60 minutes and days with triptan use were considered migraine days. Consecutive migraine days were considered as one attack. Attacks that were temporarily remitted and recurred within 48 hours were also considered as one attack.

To distinguish actual menstruation from spotting and erroneous entries, the median menstrual cycle length was calculated for each patient. Menstrual cycles that were shorter than the patients median or 21 days, were considered as potential erroneous entries. A script was developed to check these entries and if possible reset them in such a way that the menstrual cycle length became as close as possible to a minimum of 21 days or the patients median, without exceeding a maximum length of 35 days or the patients median. The duration of the menstrual bleeding was also taken into account. Longer menstrual bleedings were considered to represent menstruation more reliably than menstrual bleedings lasting only one day. If women reported only single days of blood loss, the menstrual cycle length alone was used to distinguish menstrual bleedings from spotting.

For each patient ICHD-3 criteria for MM were verified in the diary, i.e. DB ICHD-3, which was defined as the occurrence of a perimenstrual migraine attack in two out of three consecutive menstrual cycles (14). A perimenstrual migraine attack was defined as an attack starting on day 1 ± 2 of the menstrual cycle (day −2 till +3, there is no day 0). Attacks that were already ongoing before day −2 were considered non-perimenstrual. In women using hormonal contraception, withdrawal bleeding was considered as menstruation, while breakthrough bleedings were not (14). A distinction between menstrually-related and pure MM was made based on the presence of non-perimenstrual attacks. Diagnosis was based on the first three menstrual cycles registered in the E-diary.

Statistical MM was additionally assessed using a mathematical model (17). For each woman a 2 × 2-contingency table was created with the number of perimenstrual and non-perimenstrual days, and the number of migraine attacks and days where a migraine attack could have started. The 48 hours following a migraine attack were disregarded, as start of an attack during this interval would be interpreted as a continuation of the same attack. Subsequently, one-sided Fisher Exact tests were applied with mid-p correction, and women with p-values <0.1 were diagnosed with sMM (17). Diagnosis was based on all available E-diary data.

Validity of self-reported MM compared to both DB ICHD-3 diagnosis and sMM was assessed by calculating the sensitivity, specificity, positive predictive value (PPV) and negative predictive value (NPV).

Test-retest reliability of DB ICHD-3 and sMM was assessed in patients who were followed during at least six menstrual cycles, by comparing the diagnosis of menstrual cycles 1–3 and cycles 4–6. Furthermore, concordance between DB ICHD-3 diagnosis and sMM was evaluated with Cohen’s kappa and two-way contingency tables of frequencies.

Migraine incidence was calculated and plotted for each day of the menstrual cycle for women with a natural menstrual cycle and women using hormonal contraception. In both groups, day 1 was defined as the first day of the menstrual bleeding or withdrawal bleeding. Menstrual cycles were standardized to 28 days; the perimenstrual days of the menstrual cycle were fixed to 5 days, while the non-perimenstrual days were standardized to 23 (28–5) days. In addition, plots were made for women with and without DB ICHD-3 and sMM diagnosis.

Lastly, to assess whether DB ICHD-3 and sMM diagnosis enabled identifying women with more severe migraine attack characteristics during the menstrual window, we compared clinical characteristics of perimenstrual and non-perimenstrual migraine attacks for women with and without MM diagnosis. The following outcome measures were considered: attack duration in hours, triptan use and recurrence of a migraine attack within 48 hours after triptan intake. CM, MOH and use of hormonal contraception were considered potential confounders and therefore included as covariates in all statistical models. Difference in attack duration was assessed with a linear mixed effects model with the relation between the migraine attack and menstruation and potential confounders as fixed effects, and the patient as a random effect. Attack duration was log transformed to achieve a normal distribution. Differences in triptan use and recurrence between perimenstrual and non-perimenstrual attacks were analysed with logistic mixed effects models in a similar fashion. Post hoc subgroup analyses were performed in women with a natural menstrual cycle and women using hormonal contraception.

All analyses were performed in R version 4.0.5. Two-sided p-values <0.05 were considered statistically significant.

## Results

**Figure 1.**
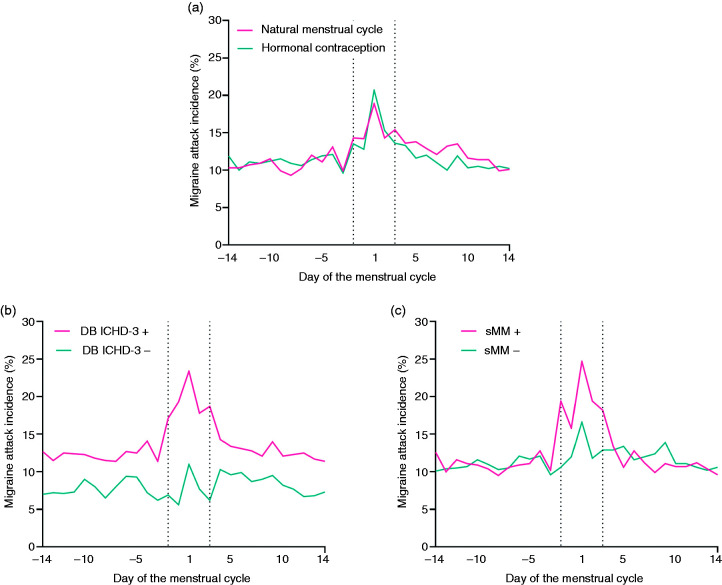
(a) Migraine attack incidence on each day of the menstrual cycle for women with a natural menstrual cycle and women using hormonal contraception. In both groups day 1 of the menstrual cycle was defined as the first day of bleeding. Menstrual cycles were standardized to 28 days; the perimenstrual days of the menstrual cycle were fixed to 5 days, while the non-perimenstrual days were standardized to 23 (28–5) days. (b) Migraine incidence on each day of the menstrual cycle for women with and without menstrual migraine according to DB ICHD-3 diagnosis. (c) Migraine incidence on each day of the menstrual cycle for women with and without menstrual migraine according to sMM diagnosis.

Headache E-diaries were collected during ≥3 menstrual cycles for 607 women, of whom 404 had a natural menstrual cycle and 203 used hormonal contraception. Median follow-up time was 84 (IQR 84?223) days. Median number of menstrual bleedings during follow-up was 4.0 (IQR 3.0?6.0) with a median cycle length of 28.0 (IQR 26.5?30.0) days. Median menstrual cycle length in women using hormonal contraception was 33.0 (IQR 28.0–54.1) days. All women were using combined oral contraceptives including hormone free intervals, but the frequency of the hormone free intervals varied between patients; 54 women followed a 21/7 regimen, all others followed an extended regimen. Median menstrual cycle length in women with a natural menstrual cycle was 27.0 (IQR 25.7–28.5) days. In total, 15.7% of women suffered from CM and 15.0% from MOH. Baseline characteristics for both groups are presented in Table 1. Women with a natural menstrual cycle were older and median menstrual cycle length was longer in women using hormonal contraception. Figure 1A shows the migraine incidence on each day of the menstrual cycle for both groups.

**Table 1.** Baseline characteristics for women with a natural menstrual cycle and women using hormonal contraception.

	Natural menstrual cycle	Hormonal contraception
Number of patients, n	404	203
Age (years), mean ± SD	39.7 ± 7.6	33.1 ± 9.8
Migraine with aura, n (%)	148 (36.6)	79 (38.9)
Cycle length (days), median ± IQR	27.0 ± 2.8	33.0 ± 26.1
Number of cycles, median ± IQR	3.0 ± 2.0	4.0 ± 3.0
Migraine days/month, median ± IQR	5.0 ± 4.6	5.0 ± 5.1
Chronic migraine, n (%)	64 (15.8)	31 (15.3)
Medication overuse headache, n (%)	60 (14.9)	31 (15.3)
Follow-up duration (months), median ± IQR	3.0 ± 2.0	7.0 ± 10.0
E-diary compliance (%), median ± IQR	99 ± 4	98 ± 5

Chronic migraine was defined according to the ICHD-3 criteria as 3 consecutive months with ≥15 headache days and ≥8 migraine days per month (14). Medication overuse headache was defined as 3 consecutive months with ≥10 acute medication days per month (≥10 for triptans, ≥15 for analgesics, ≥10 for combination of acute medications) (14). A month was defined as a period of 28 days.

### Self-reported MM

At baseline, 83% (502/607) of women reported suffering from MM, and 7% (44/607) from pure MM. Women with a natural menstrual cycle were more likely to report an association between their migraine and menstruation than women using hormonal contraception (90% vs 69%, p < 0.001).

### DB ICHD-3 diagnosis

From the total cohort, 66% (403/607) of women fulfilled DB ICHD-3 diagnostic criteria for MM and 0.3% (2/607) for pure MM. When the two out of three criterion was released for pure MM, i.e. women with only one perimenstrual attack in three menstrual cycles, 0.8% (5/607) of women fulfilled criteria. Prevalence of MM (65% vs 68%, p = 0.425) and pure MM (0.2% vs 0.5%, p = 1.0) did not differ between women with a natural menstrual cycle and women using hormonal contraception.

### sMM diagnosis

Applying the mathematical method yielded 175/607 (29%) women diagnosed with sMM, of whom 102 had a natural menstrual cycle and 73 used hormonal contraception. Prevalence of sMM could not be compared between the two groups due to the difference in follow-up time (Table 1).

### Accuracy of self-reported MM

Frequencies of self-reported MM compared to DB ICHD-3 diagnosis and sMM are tabulated in Table 2. Self-reported MM had a sensitivity of 85%, specificity of 21%, PPV of 68% and NPV of 42% compared to DB ICHD-3 diagnosis, and a sensitivity of 83%, specificity of 18%, PPV of 29% and NPV of 72% compared to sMM. As not all patients received the E-diary directly after estimating the relationship between their migraine and menstruation, a sensitivity analysis was performed in n = 424, for whom the E-diary was started immediately afterwards. Results were very similar with a sensitivity of 89%, specificity of 20%, PPV of 67% and NPV of 49% (DB ICHD-3 as golden standard).

**Table 2.** Validity of self-reported menstrual migraine diagnosis with (A) DB ICHD-3 criteria as golden standard and (B) sMM as golden standard. DB ICHD-3 diagnosis was based on the first three menstrual cycles registered in the headache E-diary. The statistical model was applied on all available E-diary data.

	DB ICHD-3 +	DB ICHD-3 −	
A

Self-reported +	340	162	502
Self-reported −	61	44	105
	401	206	607
*Sensitivity 85%, specificity 21%, PPV 68% and NPV 42%*			

B	sMM +	sMM −	
Self-reported +	146	356	502
Self-reported −	29	76	105
	175	432	607
*Sensitivity 83%, specificity 18%, PPV 29% and NPV 72%*			

### Test-retest reliability and concordance

**Figure 2.**
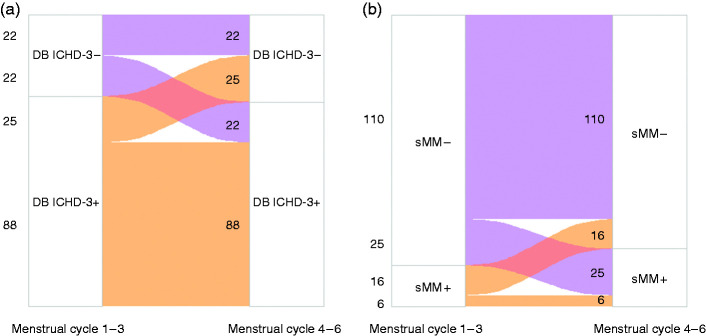
Test-retest reliability of (a) DB ICHD-3 diagnosis and (b) sMM for the first three (1–3) and following three (4–6) menstrual cycles.

A total of 157 women (90 with a natural menstrual cycle and 67 using hormonal contraception) were followed for ≥6 menstrual cycles. Test-retest reliability of DB ICHD-3 diagnosis between menstrual cycles 1–3 and menstrual cycles 4–6 was 70% (110/157), of which 80% was consistently diagnosed with MM and 20% was consistently not (Figure 2A).

Test-retest reliability of sMM diagnosis was 74% (116/157), of which 5% was consistently diagnosed with sMM and 95% was consistently not (Figure 2B).

Cohen’s kappa showed minimal concordance between DB ICHD-3 diagnosis and sMM (κ = 0.28, 95% CI: 0.23–0.33). The 2 × 2-contingency table of frequencies for DB ICHD-3 diagnosis and sMM is presented in Table 3A. Sensitivity of sMM was 41%, specificity 95%, PPV 94% and NPV 45% compared to DB ICHD-3. After exclusion of women with CM, concordance between DB ICHD-3 and sMM remained minimal (κ = 0.30, 95% CI: 0.25–0.36). The 2 × 2-contingency table is presented in Table 3B.

**Table 3.** Concordance between DB ICHD-3 diagnosis and sMM in the entire cohort (A) and in women without chronic migraine (B). The DB ICHD-3 diagnosis was based on the first 3 menstrual cycles registered in the headache E-diary. The statistical model was applied on all available E-diary data.

	DB ICHD-3 +	DB ICHD-3 −	
A
sMM +	164	11	175
sMM −	237	195	432
	401	206	607
*Sensitivity 41%, specificity 95%, PPV 94% and NPV 45%*			
B			
sMM +	135	9	144
sMM −	190	178	368
	325	187	512
*Sensitivity 42%, specificity 95%, PPV 94% and NPV 48%*			

### Women with versus without DB ICHD-3 diagnosis

Migraine incidence for each day of the menstrual cycle for women with and without DB ICHD-3 diagnosis is shown in Figure 1B. Women with DB ICHD-3 diagnosis more often suffered from CM (17.9% vs 9.3%, p = 0.009).

Clinical differences between perimenstrual and non-perimenstrual migraine attacks in women with and without DB ICHD-3 diagnosis are shown in Table 4. After correction for potential confounders, perimenstrual attack duration was 37% longer than non-perimenstrual attack duration in women with DB ICHD-3 diagnosis, while no significant differences were found in women without the diagnosis. Women with DB ICHD-3 diagnosis were more likely to take triptans during perimenstrual attacks than non-perimenstrual attacks (OR: 1.19, 95%CI: 1.09–1.31, p < 0.001), in contrast to women without DB ICHD-3 diagnosis (OR: 0.80, 95%CI: 0.64–1.00, p = 0.053). In both women with (OR: 2.66, 95%CI: 2.29–3.10, p < 0.001) and without (OR: 2.18, 95%CI: 1.50–3.18, p < 0.001) DB ICHD-3 diagnosis, perimenstrual migraine attacks were more likely to recur within 48 hours after triptan intake. Post hoc subgroup analyses in women with a natural menstrual cycle revealed similar results. In women using hormonal contraception a trend in the same direction was found, but the differences in attack duration between women with and without DB ICHD-3 diagnosis did not reach statistical significance.

**Table 4.** Differences between perimenstrual and non-perimenstrual migraine attacks in women with and without DB ICHD-3 diagnosis.

	DB ICHD-3+ (n = 401 women)				DB ICHD-3− (n = 206 women)				
	Perimenstrual migraine days (n = 4751)	Non-perimenstrual migraine days (n = 14114)	Adjusted ratio of means/odds ratio (95% CI)	Adjusted p-value	Perimenstrual migraine days (n = 716)	Non-perimenstrual migraine days (n = 5201)	Adjusted ratio of means/odds ratio (95% CI)	Adjusted p-value	Adjusted p-value for difference between DB ICHD-3+ versus DB ICHD-3−
Duration (hours), median (IQR)	24.7 (13.2–42.2)	17.0 (10.4–28.7)	1.37 (1.31–1.44)	<0.001	15.6 (6.9–32.9)	16.5 (9.0–28.5)	1.12 (1.00–1.25)	0.054	<0.001
Use of triptans, n (%)	2235 (47.0%)	6175 (43.8%)	1.19 (1.09–1.31)	<0.001	297 (41.5%)	2024 (38.9%)	0.80 (0.64–1.00)	0.053	<0.001
Recurrence<48 hours, n (%)	445 (25.0%)	1520 (22.5%)	2.66 (2.29–3.10)	<0.001	57 (22.5%)	348 (16.4%)	2.18 (1.50–3.18)	<0.001	0.41

Intra-individual means were calculated for perimenstrual attacks and non-perimenstrual attacks prior to group calculations to account for the correlation between migraine attacks within the same woman. Numbers of attacks included in the analyses on duration were n = 1972, n = 7560, n = 356 and n = 2046 respectively. Prevalence of triptan use was calculated based on the number of migraine days (column titles), while recurrence was calculated based on the number of migraine days with triptan intake including 48 hours (n = 1777, n = 6763, n = 253 and n = 2122, respectively). Note: the adjusted odds ratio and p-values are calculated with a mixed effects model corrected for potential confounders.

### Women with versus without sMM

Migraine incidence for each day of the menstrual cycle for women with and without sMM is shown in Figure 1C. Women with sMM had a longer median follow-up duration than women without sMM (142 ± 267 vs 84 ± 84 days, p < 0.001). When sMM diagnosis was based on the first three menstrual cycles 14% of women could be diagnosed. When the total follow-up duration was taken into account, 29% was diagnosed with sMM.

Clinical differences between perimenstrual and non-perimenstrual migraine attacks in women with and without sMM are shown in Table 5.

**Table 5.** Differences between perimenstrual and non-perimenstrual migraine attacks in women with and without sMM diagnosis.

	sMM + (n = 172 women)				sMM− (n = 406 women)				
	Perimenstrual migraine days (n = 2784)	Non-perimenstrual migraine days (n = 7042)	Adjusted ratio of means/odds ratio	Adjusted p-value	Perimenstrual migraine days (n = 2683)	Non-perimenstrual migraine days (n = 12273)	Adjusted ratio of means/odds ratio	Adjusted p-value	Adjusted p-value for difference between sMM+ versus sMM−
Duration (hours), median (IQR)	27.0 (17.0–44.2)	17.0 (10.3–28.7)	1.50 (1.40–1.61)	<0.001	19.2 (8.7–37.0)	16.9 (9.8–28.7)	1.19 (1.12–1.27)	<0.001	<0.001
Use of triptans, n (%)	1354 (48.6%)	3129 (44.4%)	1.22 (1.08–1.37)	0.001	1178 (43.9%)	5070 (41.3%)	1.04 (0.92–1.18)	0.51	0.08
Recurrence <48 hours, n (%)	202 (22.1%)	816 (22.6%)	2.56 (2.03–3.22)	<0.001	300 (26.9%)	1052 (20.0%)	2.64 (2.21–3.16)	<0.001	0.58

Intra-individual means were calculated for perimenstrual attacks and non-perimenstrual attacks prior to group calculations to account for the correlation between migraine attacks within the same woman. Numbers of attacks included in the analyses on duration were n = 1131, n = 3716, n = 1197 and n = 5890 respectively. Prevalence of triptan use was calculated based on the number of migraine days (column titles), while recurrence was calculated based on the number of migraine days with triptan intake including 48 hours (n = 914, n =  3612, n = 1116 and n = 5273, respectively). Note: the adjusted odds ratio and p-values are calculated with a mixed effects model corrected for potential confounders.

## Discussion

The aim of this study was to assess validity of the current ICHD-3 appendix criteria for MM. Based on our results, we have formulated some recommendations and considerations for diagnosing MM.

Firstly, according to the ICHD-3 appendix no headache diaries are necessary for a clinical MM diagnosis. However, our results indicate that the accuracy of self-reported MM is extremely poor. Both women who do and women who do not report an association between their migraine and menstrual cycle fulfil diagnostic criteria in two thirds of cases. These results are in line with earlier studies, which have demonstrated that migraine patients experience difficulties recalling information about their clinical condition, such as estimating their monthly attack frequency and intake of acute medication, and also specifically in estimating the relation to the menstrual cycle (15,16,21). We strongly recommend the use of prospective headache (E-)diaries instead of simply asking patients, both for research purposes and in clinical practice.

Based on our results, we suspect that pure MM is rare (< 1%). A distinction between menstrually-related and pure MM may thus be of limited clinical relevance in a hospital or research population (as women with only few migraine attacks per year may not seek medical attention or participate in research) and leaving pure MM out of future ICHD criteria may be considered.

Both DB ICHD-3 diagnosis and sMM seem to be able to distinguish women who are more severely affected during the perimenstrual window from women who are not. Women with DB ICHD-3 MM have a longer attack duration and increased triptan intake during perimenstrual migraine attacks, which also corresponds to what is known about MM so far (2,3,22,23). While earlier studies have mainly focused on differences between perimenstrual attacks and non-perimenstrual attacks in women with MM, we have also assessed and compared differences in women without MM. We found smaller or no differences in attack duration and triptan intake between perimenstrual and non-perimenstrual attacks in women without MM, indicating that the DB ICHD-3 method identifies a subgroup of women who suffer from longer and more refractory migraine attacks during the menstrual window. Post hoc subgroup analyses suggest that these results apply to both women with a natural menstrual cycle and women using hormonal contraception, although not all outcomes within the subgroup of women using hormonal contraception reached statistical significance. Surprisingly, we did find an increased recurrence risk during the perimenstrual period, also in women without MM. This could be explained by false negative diagnoses, but an alternative explanation may be that hormonal changes prior to the menstruation have an influence on all women with migraine, although in a varying degree.

Applying DB ICHD-3 criteria yields more women diagnosed with MM than application of the sMM criteria (66% vs 29%). Both methods have a test-retest reliability of approximately 70%. Physicians and researchers should be aware that, if they diagnose a patient based on three menstrual cycles, in 25–30% of cases the diagnosis may be different in the following three cycles.

Another disadvantage of the DB ICHD-3 diagnosis is the potential overrepresentation of women with a high attack frequency and underrepresentation of women with a low attack frequency. We have shown that women with a DB ICHD-3 diagnosis have a higher number of monthly migraine days and more often suffer from CM. Whether it is appropriate to diagnose women with CM with MM is disputable and requires further research. However, we hypothesize that, as perimenstrual migraine attacks are of longer duration and repeated triptan intake is often necessary, women with MM may be at increased risk for medication overuse headache and conversion to CM.

The sMM method takes the ratio between perimenstrual and non-perimenstrual migraine attacks into account, which might partially solve the problem that with higher number of attacks per month it is more likely that one of these attacks occurs during the menstrual window. However, disadvantages of sMM are its complexity, i.e. implementation of statistical analyses in clinical practice, and its demand for a relatively long follow-up period to achieve the required statistical power. When the method was applied to three menstrual cycles, only 14% of women could be diagnosed with sMM, compared to 29% when all available E-diary data were taken into account. In clinical practice, but also in research, a baseline period of more than three menstrual cycles is impractical and thus undesirable.

The current study has some major strengths. Firstly, data were collected in a large and well-defined patient population during a relatively long follow-up period. We used our previously developed and validated headache E-diary, which provided us with daily information about the occurrence of (migraine) headache and menstruation (15). The definition of a migraine day was based on an automatic algorithm according to ICHD-3 criteria (14). The questionnaires were time-locked after 48 hours, meaning that patients could only provide recent information, reducing the chance of recall bias. Patients were encouraged to fill out information each day and not only on headache days in order to gain a complete overview. No patients had to be excluded due to <80% compliance.

A limitation of the current study may be that patients were in part recruited from our tertiary headache center and therefore the number of mean monthly migraine days was relatively high. In addition, women with CM were not excluded, although it may be disputed whether women with CM can be diagnosed with MM due to the likelihood of chance diagnosis (17). In our cohort, prevalence of CM was comparable between women with and without sMM (18% vs 15%), while a relative overrepresentation of CM was found in women with DB ICHD-3 (19% vs 9%). Performance of DB ICHD-3, and to a lesser extent sMM, could thus differ in patients with a lower attack frequency, and therefore results may not be entirely generalizable to all female migraine patients.

Selecting women with a higher than chance association between migraine and the menstrual cycle can be a challenge. We advise caregivers and researchers not to simply ask women about the relationship between migraine and the menstrual cycle retrospectively, but to collect prospective headache (E-)diaries during at least three menstrual cycles with application of the ICHD-3 criteria. For research purposes, application of sMM criteria may provide more certainty about the robustness of the relationship, but a longer follow-up period may be required which makes it more impracticable

For future editions of the ICHD, we suggest to keep MM in the appendix, as there is not one perfect method to define MM. In addition, we recommend (E-)diary based diagnosis for both clinical practice and research purposes. Lastly, we propose to omit the distinction between menstrually-related and pure MM, and only provide criteria for menstrual migraine (Table 6).

**Table 6.** Proposal for menstrual migraine diagnostic criteria in future editions of the ICHD Appendix (14).

	Diagnostic criteria
A	Attacks, in a menstruating woman^1^, fulfilling criteria for migraine with or without aura
B	Starting on day 1 ± 2 (ie, days −2 to +3)^2^ of menstruation^1^ in at least two out of three menstrual cycles^3,^^4^

	Notes
1.	Menstruation is considered to be endometrial bleeding resulting either from the normal menstrual cycle or from the withdrawal of exogenous progestogens, as in the use of combined oral contraceptives or cyclical hormone replacement therapy.
2	The first day of menstruation is day 1 and the preceding day is day −1; there is no day 0.
3	Attacks may occur additionally at other times of the cycle.
4	A prospective (E-)diary is strongly recommended for both research purposes and clinical practice.

## Article highlights

- Self-reported menstrual migraine diagnosis has extremely poor accuracy. Both women who do and women who do not report an association between their migraine and menstrual cycle fulfil diagnostic ICHD-3 criteria in two third of cases.
- Prospective headache (E-)diaries are required for a reliable menstrual migraine diagnosis, also in clinical practice.
- Pure MM is extremely rare and a distinction between menstrually-related MM and pure MM may therefore be of limited clinical relevance.
- In women diagnosed with menstrual migraine, perimenstrual attacks are of longer duration and associated with increased triptan intake compared to non-perimenstrual attacks, while no such differences are found in women with non-menstrual migraine. In both groups perimenstrual attacks are more likely to recur than non-perimenstrual attacks.
